# Fully Printed Flexible Single-Chip RFID Tag with Light Detection Capabilities

**DOI:** 10.3390/s17030534

**Published:** 2017-03-08

**Authors:** Aniello Falco, Jose F. Salmerón, Florin C. Loghin, Paolo Lugli, Almudena Rivadeneyra

**Affiliations:** 1 Institute for Nanoelectronics, Technical University of Munich, 80333 Munich, Germany; jf.salmeron@tum.de (J.F.S.); florin.loghin@tum.de (F.C.L.); almudena.rivadeneyra@tum.de (A.R.); 2 Faculty of Science and Technology, Free University of Bolzano, 39100 Bolzano-Bozen, Italy; Paolo.Lugli@unibz.it

**Keywords:** flexible substrate, inkjet printing, organic photodetector, printed electronics, spray deposition, OPD

## Abstract

A printed passive radiofrequency identification (RFID) tag in the ultra-high frequency band for light and temperature monitoring is presented. The whole tag has been manufactured by printing techniques on a flexible substrate. Antenna and interconnects are realized with silver nanoparticles via inkjet printing. A sprayed photodetector performs the light monitoring, whereas temperature measurement comes from an in-built sensor in the silicon RFID chip. One of the advantages of this system is the digital read-out and transmission of the sensors information on the RFID tag that ensures reliability. Furthermore, the use of printing techniques allows large-scale manufacturing and the direct fabrication of the tag on the desired surface. This work proves for the first time the feasibility of the embedment of large-scale organic photodetectors onto inkjet printed RFID tags. Here, we solve the problem of integration of different manufacturing techniques to develop an optimal final sensor system.

## 1. Introduction

The increasing interest in the Internet of things (IoT) has opened a new paradigm in the interaction between objects and human beings. In the IoT, sensors and actuators are embedded in physical objects and interconnected through wired and wireless networks [1]. One of the existing alternatives to connect objects is the radiofrequency identification (RFID) technology. RFID tags with sensing capabilities can be attached to objects, allowing not only their identification at different locations but also obtaining information about their quality or the environment where they are placed. In the case of products, it is especially interesting to know its status through the supply chains, leading to improvements in the inventory management as well as reductions in working capital and logistics costs [2]. This situation has led to a new paradigm in packaging [3].

Different environmental parameters are meaningful to control, such as relative humidity, temperature or exposure to certain chemicals [4,5,6]. European studies estimates that 40% to 50% of all food produced in Europe is wasted, where 10% to 15% of this wastage happens during supply and delivery operations to consumers [7]. Furthermore, the food industry disposed about $25 billion of spoiled goods every year. In addition to this, within every home, annually $300 worth of bruised fruit, bad meat, and other perishable goods are thrown away [7]. This situation has severe consequences in the global supply of adequate food stocks and contributes to escalating food costs. In particular, the exposure to light and its dose is a very significant environmental factor influencing the quality and lifetime of perishables [8]. Apart from that, light controlling can be used as a security measure, detecting if a package has been opened or badly manipulated. Some strategies have been followed, such as the inclusion of a tape that can be used to seal the package. This tape consists of two parallel conductive lines connected by conductive bridges that has to be broken to open the package, thus allowing the detection of any intrusion into the package [9].

In this regard, printed electronics (PE) results are especially attractive to develop these kinds of solutions. Printing has the capability of manufacturing over large areas with ultra-cost-effectiveness. Other relevant features of PE are the capabilities of achieving lightweight, thin, and environmental-friendly devices [10]. Different techniques can be used to fabricate RFID tags with PE, from screen-printing to inkjet printing or gravure; furthermore, the tag can be fabricated directly on the desired substrate, such as paper or plastic substrate. Not only the antenna and the connections can be manufactured by PE [11,12], but also the sensor capabilities can be developed with these techniques [13,14,15]. Many sensors useful for packaging monitoring have been already developed [16]. It is crucial to minimize the number of discrete components that are integrated into these types of devices in order to reduce its cost per unit. To do that, the inclusion of printed sensors and antennas is mandatory.

In this work, we present a novel ultra-high frequency (UHF) RFID tag developed on a flexible substrate including a photodetector. Some silicon solutions have been already developed in this direction [17,18], but the development of a flexible and printed one has not been described until now. This tag has been fully fabricated by printing techniques, except for the silicon RFID chip, showing the strategy followed to develop a final working device making profit of different manufacturing techniques. In particular, the antenna and layout have been inkjet printed while the photodiode structure has been realized by spray deposition. Indeed, the major challenge of this work is to successfully fabricate the sprayed photodetector on a flexible substrate, using as the bottom electrode an inkjetted silver line. We present the difficulties of the fabrication integration and how we overcome them. The selected RFID chip includes a sensor front-end that is able to read out the response of the photodetector without requiring any other external component. In addition, an in-built temperature sensor is implemented in the chip, increasing the functionalities of this tag. First, we will present the design of the tag, followed by the characterization of its antenna and photodetector. Finally, the full operation capabilities of the RFID tag will be shown. This tag can be used to control the exposure of products to light, not only as a threshold sensor but also to know the absorbed light dose. Furthermore, by including the RFID tag in the inner part of a packet, any anomaly in the closure of the packet or if it has been opened is registered with high accuracy by the sensor when a reader is in its vicinity.

## 2. Materials and Methods

### 2.1. Tag Design

**Figure 1:**
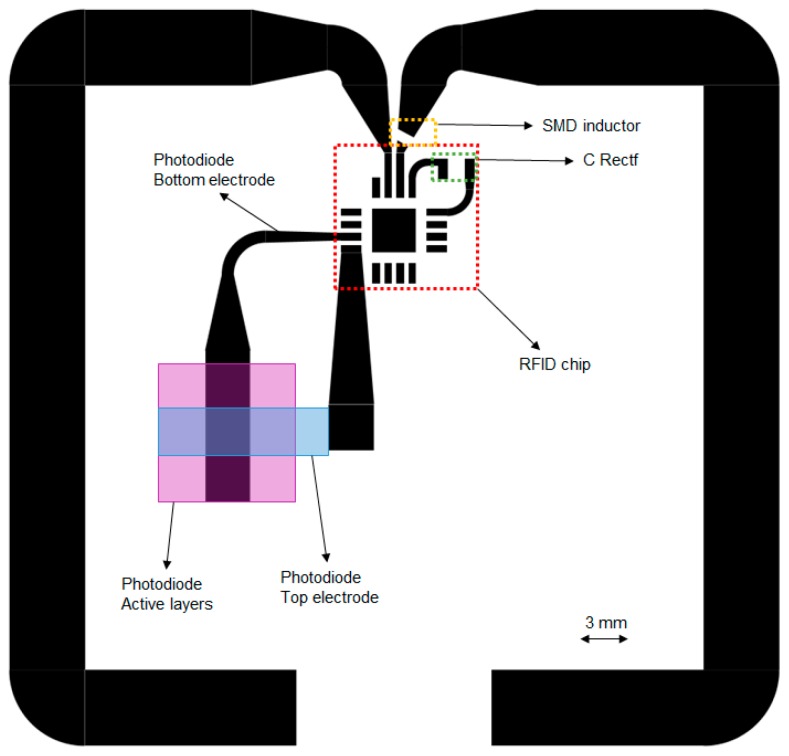
Layout of the radiofrequency identification (RFID) tag. The folded dipole encases a square of 5 × 5 cm^2^, and the width of the arms is 5 mm. The active area of the Organic Photodiode is given by the overlapping of a bottom Ag line and a top conductive polymer line, both with a width of 3 mm, with a total surface of 9 mm^2^.

Figure 1 illustrates the layout of the fabricated RFID tag, including the footprints of the different elements included. This passive tag working at the UHF band includes the RFID chip compatible with Electronic Product Code (EPC) Generation 2 RFID standard [19]. This RFID chip was selected because it integrates a sensor front-end (SFE) with different sensor conditioning stages and a 10-bit Analog to Digital Converter (ADC). In the presented work, no extra circuitry is required to interface the sensor. Furthermore, this RFID chip has an in-built temperature sensor.

Advanced Design System 2013 (Keysight Technologies Inc., Santa Rosa, CA, USA) was used to design the printed antenna and the matching network. The antenna impedance must be a complex conjugate of the chip impedance to maximize the power transfer between the antenna and the chip [20]. The RFID chip input impedance was taken from our previous work [11] and its value is (31 − j286) Ω. A dipole antenna resonating at the European UHF RFID band defines the radiofrequency interface. First, we designed the antenna resonating at 868 MHz with its real part matching the real part of the RFID chip impedance. Finally, as a matching network, one RF Surface Mount Device (SMD) inductor in series on one of the dipole arms was needed to provide the necessary large inductive part [20]. This SMD inductor could be avoided by changing the antenna layout (i.e., bow-tie antenna [11]), but we decided to include it in order to reduced area and materials. The tag has been designed to occupy a total area of 5 × 5 cm^2^. An external capacitor of 2.2 µF (C_Rectf_ in Figure 1) has been placed between the RF rectifier output (V_Rectf_) and the RFID chip ground terminal. The purpose of this component is to smooth the signal coming from the RF rectifier and, in this way, extend the maximum read range, as indicated by the manufacturer [19], but it is not mandatory.

**Figure 2:**
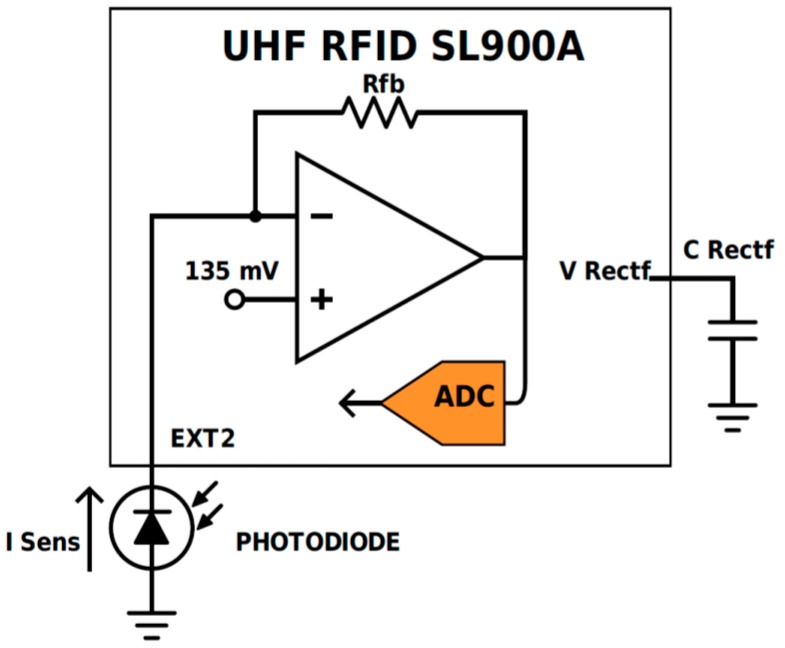
Schematics of the Sensor Front-End (SFE) configuration.

As already mentioned, a temperature sensor is integrated into the chip. Two internal voltage references set the lower and upper limits of this converter, and, therefore, a concrete resolution and range are selected. The minimum resolution of this sensor is 0.18 °C in a range from −89.3 °C to 84.6 °C, and 0.23 °C in the widest range of temperature (from −89.3 °C to 147.9 °C). A fully printed organic photodiode (OPD) is integrated into this tag. The photodetector is directly connected to the SFE of the RFID chip. To read out a current value, it is not required to have any external component. This RFID chip has been previously used with a commercial photodetector [11]. Figure 2 illustrates the sensor configuration mode selected for the read-out of the OPD, including the conditioning stage in the RFID chip.

This configuration is suitable for measuring the photocurrent generated by photodiodes. An operational amplifier with negative feedback is combined with a selectable feedback resistor (*R_fb_*) (185, 400, 875, 1875 or 3875 kΩ) (see Figure 2). The non-inverting input is fixed to 135 mV, so the input voltage in the 10 bits ADC is related to *I_Sens_* value by:

(1) $$V_{ADC}=135mV+I_{Sens}\cdot R_{fb}.$$

Two internal voltage references, V_ref1_ and V_ref2_ individually selectable in steps of 50 mV between 160 and 610 mV, set the lower and upper limits of the ADC. These limits are defined as 2 × V_ref1_ − V_ref2_ and V_ref1_. The difference between them defines the input voltage range, V_ref2_ − V_ref1_, and the limits of operation. For optimal operation, the feedback resistor value and the ADC reference voltages can be optimized for sensing a concrete range of current. In order to read out the fabricated smart tag by an RFID reader, several EPC Gen 2 custom commands must be sent to configure different parameters and read each available sensor [19]. Battery or some harvesting mechanism apart from RFID antennas would be necessary in order to perform logging operations if the tag is not in the vicinity of an RFID reader, although, at different points of the supply chain, the sensor information can be acquired by an RFID reader and stored in the internal Electrically Erasable Programmable Read-Only Memory (EEPROM) including a timestamp. In this way, logging would be possible and this information would be available for future access, e.g., by final users.

### 2.2. Materials

The substrate is a polyethylene naphthalate (PEN) (Teonex^®^ Q65H from Teijin DuPont Films Japan Limited, Tokyo, Japan) with a thickness of 125 µm. No pre-treatment of the surface is needed before manufacturing. The antenna and layout have been made of silver ink (DGP 40LT-15C from ANP USA, Inc., Pleasanton, CA, USA) with about 30% of silver nanoparticles and triethylene glycol monoethyl ether (TMGE) as the solvent.

The bulk heterojunction has been obtained dissolving regioregular poly(3-hexylthiophene-2,5-diyl) (Rieke Metals Inc., Lincoln, NE, USA) and [6,6]-phenyl C61 butyric acid methyl ester (PCBM) (Solenne B.V.) in 1,2-Dichlorobenzene (o-DCB,Sigma-Aldrich) with a 1% wt:1% wt ratio and stirred for 2 h at 60 °C. The poly(3,4-ethylenedioxythiophene) polystyrene sulfonate (PEDOT:PSS, CLEVIOS P VP CH 8000) solution was sprayed in a dilution of 1:3 in Isopropyl alcohol (Sigma-Aldrich). The highly conductive PEDOT:PSS utilized for the anodes was a mixture of PEDOT:PSS (CLEVIOS PH 1000) and Ethylene glycol. Finally, the polyethylenimine (PEI) solution was obtained dissolving the polymer with 0.1% wt in ethanol. The non-printed elements have been glued to the tag with conductive resin H20E (Epoxy Technology, Inc., Billerica, MA, USA) and a 50 μm-thick dry adhesive, AR Clear 8932 (Adhesives Research, Inc. Glen Rock, Pennsylvania, USA) for the chip assembly.

The non-printed elements included in the tag are the silicon RFID chip (SL900A RFID chip from AMS AG, Unterpremstaetten, Austria), an SMD inductor of 47 nH to match the antenna and a capacitor of 2.2 µF to optimize the read range, both from TE Connectivity, Ltd. (Schaffhausen, Switzerland).

### 2.3. Fabrication Process

The antenna, interconnections and bottom electrode have been defined with DMP-2831™ Dimatix printer (Fujifilm Dimatix Inc., Santa Clara, CA, USA). The substrate temperature has been fixed at 60 °C while printing. All of these elements have been manufactured with only one printed layer. A drop space of 20 μm has been settled in the printer for 40 μm-landed diameter drops followed by a curing step at 125 °C for 30 min. The error caused by the accuracy of the printing process is one extra drop, a half drop on each side of the layout, as detailed in [21]. Thus, the error will be 40 μm, which is insignificant compared to the printing dimensions of the prototype. Higher temperatures are not recommended because the glass transition temperature of this PEN film is 155 °C.

**Figure 3:**
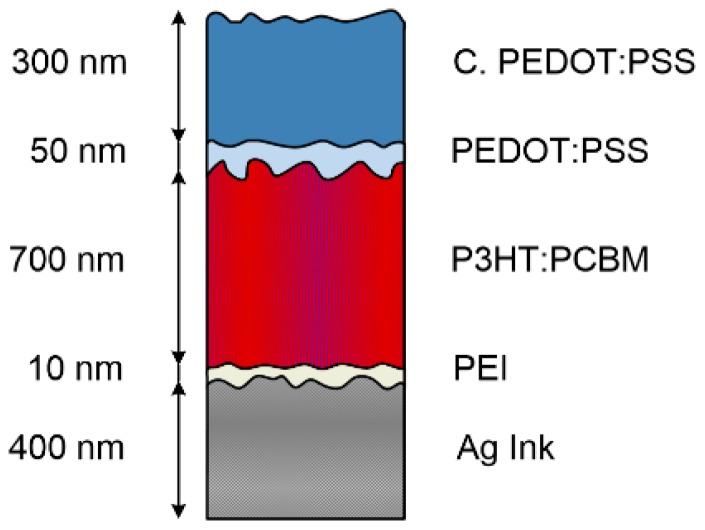
Cross-sectional view of the OPD. An inkjet printed Ag line modified with spray-coated PEI is used as reflective bottom-electrode, while a 700 nm thick blend of P3HT and PCBM is used as photoactive layer. The top semi-transparent electrode is obtained via a mixture of the conductive polymer PEDOT:PSS.

An inkjet printed line of Ag ink has been used as a cathode for the OPD. The measured work function of the Ag line, however, is too high (~4.4 eV) for the realization of functioning photodiodes and has to be lowered throughout the use of a PEI thin film, as reported in the past in literature [22,23]. Nevertheless, since the spin-coating of a PEI layer might affect the functionality of the whole printed systems, it has been necessary to realize it on a definite area via spray-deposition throughout a stencil, using the parameters developed and validated in [24]. Subsequent to the deposition of the PEI film, a 700 nm thick P3HT:PCBM film as an active layer has been sprayed, followed by a 50 nm thick hole injection layer and a 300 nm thick PEDOT:PSS anode. The fabrication parameters of these layers are presented in full detail elsewhere [25,26]. Figure 3 depicts the cross sectional view of the OPD, including its electrodes.

The spray-deposition has been carried out through self-made spray-deposition setup. An atomizing gun (Krautzberger GmbH, Eltville am Rhein, Germany) is attached to a platform with adjustable height and fixed *x*–*y* position and it is controlled by an electromechanical 3/2-way valve. The valve is activated by an electronic timer to guarantee repeatable and accurate deposition times, and the nitrogen pressure is set by a pressure gauge. The substrate temperature is adjusted via a programmable hot-plate. PEI, P3HT:PCBM and PEDOT:PSS were spray deposited at a nozzle-to-sample distance of 15 cm, with a substrate temperature of 60 °C, 120 °C and 100 °C, respectively, with an atomization pressure of 0.5 bar, 1 bar and 1 bar, respectively. These parameters were chosen as optimal in terms of roughness and substrate coverage, based on the results of previous works [24,25].

The last step has been the assembly of the chip and the external components to the flexible tag. For this purpose, a three-step process has been followed. First, interconnects between RFID chip and silver pads have been made by using the conductive resin H20E. A double layer of dry adhesive has been placed on the bottom part of the chip to fix it to the substrate. Finally, the conductive resin has been cured by heating in an oven at 80 °C for 3 h. This heating step also improves the dry film adhesion, better gluing the chip to the substrate.

### 2.4. Characterization

The antenna characterization has been performed with a ZVA40 Vector Network Analyzer (Rohde & Schwarz, Munich, Germany). The S-parameter differential port between port-1 and port-2 has been defined through a test fixture simulator included in the Vector Network Analyzer (VNA) because of the differential character of the antenna measurement. For characterization purposes, Ultra Miniature Coaxial connectors (U.FL) type (Hirose Electric, Tokyo, Japan) have been attached on the antenna feed point. SubMiniature version A (SMA)to U.FL wires have been employed to connect the antenna to the VNA, using a custom calibration kit that was developed using the U.FL connectors [11].

A commercial RFID reader compatible with EPC Gen2, DK-UHF RFID Radon (AMS AG, Unterpremstaetten, Austria) was employed to measure the read range of the tag and read out its sensor data through an application running in a PC.

The IV-characterization of the OPDs were performed by means of a Keithley 2602 sourcemeter, (Tektronix, Beaverton, OR, USA), dark current measurements were performed inside a dark chamber, while the illuminated current measurements were performed under a sun simulator at AM1.5. Finally, the External Quantum Efficiency (EQE) of the fabricated devices was evaluated using a 300 W xenon arc lamp choppered at 77 Hz, through an Oriel Cornerstone 260 ¼ m monochromator (Newport Corporation, Irvine, CA, USA) and an Oriel Merlin digital lock-in amplifier (Newport Corporation, Irvine, CA, USA). The performance of the temperature sensor was tested in the climatic chamber VCL 4006 (Vötsch Industrietechnik GmbH, Balingen, Germany).

## 3. Results and Discussion

### 3.1. Antenna Performance

**Figure 4:**
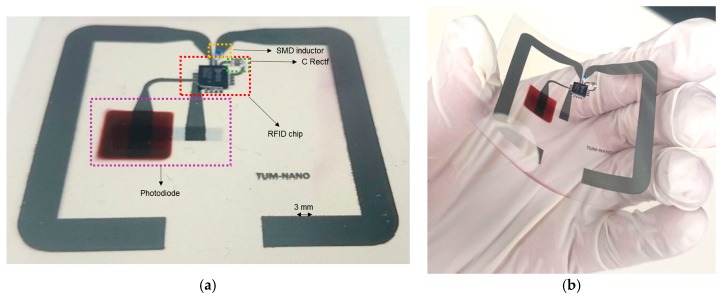
(**a**) image of the tag with all its elements labelled; (**b**) image of the tag.

Figure 4 presents the printed RFID tag including the external components, labelled for better understanding. Final dimensions of the inkjet-printed dipole antenna arms are 5 mm width and 87 mm length. The dipole arms have been bent twice to optimize the occupied area.

**Figure 5:**
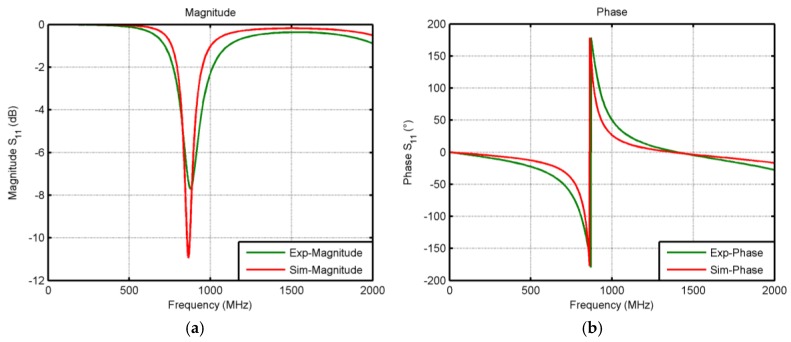
Experimentally measured (green solid line) and simulated (red solid line) magnitude (**a**); and phase (**b**) of differential S-parameter of the dipole antenna.

Figure 5 shows the S-parameter response of the dipole antenna, both simulated and measured responses. The measured resonant frequency fulfills the simulations results, although we found a difference on antenna impedances. The measured input impedance is (55 + j23) Ω, whilst he antenna impedance obtained by simulation is (31.1 + j23) Ω. This difference could be associated with the parasitic resistance of the SMD inductor and with the printed silver electrical behavior. In the Electromagnetic (EM) simulation, a silver metallic layer is modeled as bulk material, whilst the printed patterns differ from this ideal assumption, causing increases in the ohmic loss [21]. Therefore, an extra loss would be introduced in the RF link between reader and tag due to this mismatch. This drawback could be overcome optimizing the antenna design through new EM simulations or using a different matching network topology with more versatility such as a T-Network. Finally, the chosen SMD inductor has an inductance of 47 nH, size of 0603, and a quality factor of 60 at 900 MHz (SRF = 1.9 GHz).

The maximum read range, according to the theory of communication in RFID systems [20,27,28], is calculated using the following Equation (2):

(2) $$range_{\max}=\frac{\lambda }{4\pi }\sqrt{\frac{PLF\cdot G_{tag}\cdot G_{reader}\cdot P_{reader}\cdot \tau }{s_{tag}}},$$

where Stag is the RFID chip sensitivity; the minimum received power level to activate the tag is −15 dBm for the chosen RFID chip. However, in this particular case, the harvested energy from the EM field radiated by the RFID reader is necessary to retrieve the sensor information. The necessary power to drive the sensors comes from the internal RF rectifier of the RFID chip. According to the manufacturer, operating at 1.5 V in passive mode requires 200 µA to power up the chip and the internal ADC converter in charge of processing the acquired signals. Therefore, we have defined a new tag sensitivity of −2.21 dBm, which is calculated defining the RF rectifier efficiency of ~40%. G_tag_ refers to the tag antenna gain, P_reader_ is the effective radiated power by the reader and G_reader_ is the reader antenna gain.

Tag antenna parameters obtained by EM simulation are the following: gain is −0.98 dBi, directivity 1.67 dBi and efficiency 54.47% at the working frequency. The reader transmission power is 26 dBm and the reader antenna gain is 7 dBi at 868 MHz, according to the manufacturer. The polarization mismatch between the reader antenna (circular polarization) and the designed tag antenna (lineal polarization) is also included. This is represented as the polarization loss factor (PLF) in (2) and its value is 0.5 (−3 dB). The factor *τ* takes into account the losses related to the mismatch between the chip and the antenna.

The maximum read range has been only 80 cm whilst the estimated one is 1.4 m. The estimated maximum read range assumes a perfect matching between chip and antenna (*τ* = 1 in Equation (2)), but, as previously stated, the different real parts of antenna and RFID chip impedances causes a mismatching (*τ* = 0.72 in Equation (2)) that reduces the read range to 1.2 m. The reasons of the reduced read range, apart from the impedance mismatch, are related to the poor antenna gain. Both limitations are caused by the low thickness obtained with printing one single layer, according to the model presented in [21], below 0.4 µm. Several strategies can be followed to overcome this limitation like printing several layers or increasing the printing resolution [29]. Apart from that, it should be noticed that dipole antennas suffer from lower performance when their arms are bent [30,31], so the maximum reading range will be further decreased if the tag is under mechanical deformation.

### 3.2. Photodiode Characterization

Although spin-coating is a well-established technology in the fabrication of OPDs [32], it presents few major disadvantages. In a first instance, the use of spin-coating as a deposition technique prevents a large-scale uniform deposition of thin films, especially on flexible or uneven substrates such as plastics or paper. Furthermore, spin-coating is intrinsically a full-sample deposition method, and the patterning is only possible through classical photolithography, a relatively complex process which includes the use of solvents, but not always compatible with plastic and paper electronics. The usage of easy-to-pattern deposition techniques is then necessary for the fabrication of the OPDs into an integrated printed system, rendering inkjet printing [23] and spray-coating [33] as primary choices for the fabrication of integrated printed systems.

The typical material stack of an OPD includes a low work function conductor to be used as a cathode, the photoactive material and a high work function conductor to be used as an anode, and at least one of the two contacts must be semi-transparent to allow the light to penetrate into the photoactive material. Albeit the realization of semi-transparent solution-processable high work function electrodes has been demonstrated in the past with the use of conductive polymers, such as PEDOT:PSS [23,26,34], the obtainment of low work function materials is hindered by their high reactivity to oxygen. For instance, printed silver, which is characterized by a nominal work function of 4.26 eV, as a consequence of oxygen chemisorption, can show an increase in this parameter of up to 1 eV, leading to poor reproducibility and sub-optimal devices [23,35]. One of the most reliable solutions to this problem is the use of surface modifiers, such as polyethylenimine (PEI), to induce permanent dipoles, consequently reducing and stabilizing the work function of the material [22]. The PEI thin layer (around 10 nm) is typically deposited through spin-coating or dip coating, which leads to the deposition onto the whole substrate and could compromise the functionality of the other devices integrated there. Conversely, the obtainment of a spray-deposition method through a stencil guarantees the realization of a precisely patterned PEI layer, which represents the basis for the production of the stacked photodetector in a complex system.

**Figure 6:**
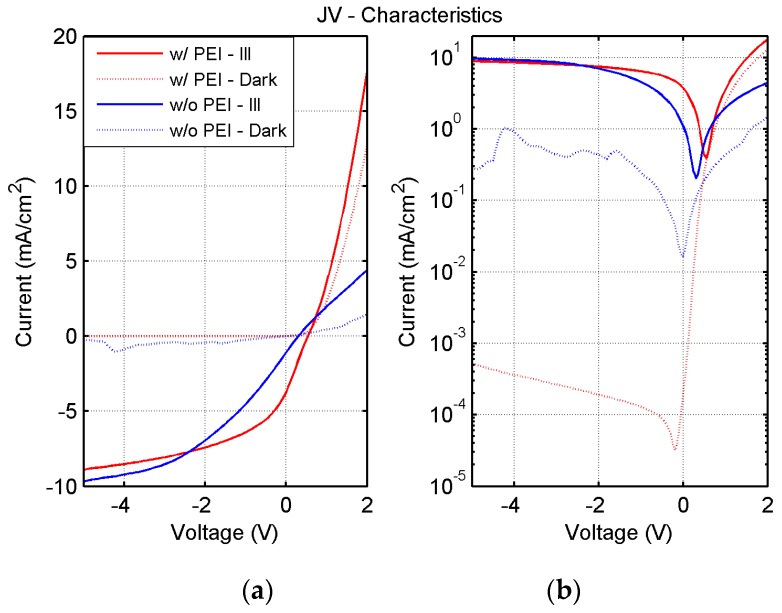
Dark (dashed lines) and illuminated (solid lines) JV characteristics, in linear (**a**) and logarithmic (**b**) scale, of the fully-sprayed organic photodiode with and without PEI. Current density as low as 5 × 10^−4^ mA/cm^2^ have been reliably achieved.

**Figure 7:**
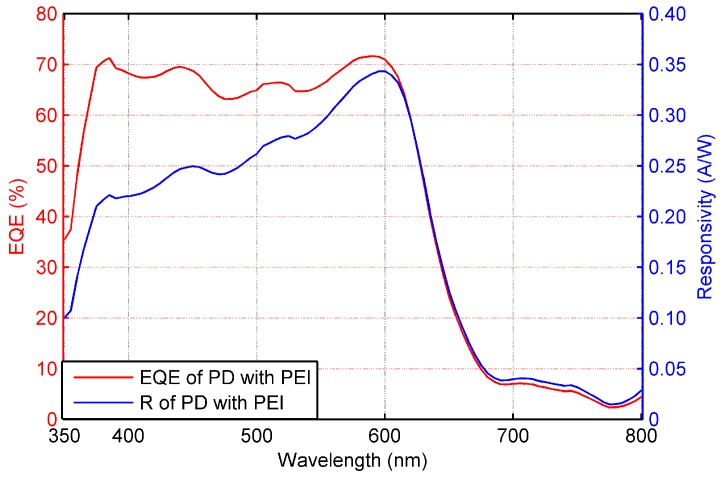
**External Quantum Efficiency** (EQE) of the OPD fabricated with the PEI interlayer. In the visible range, peak EQE of 70% and responsivity up to 0.34 A/W are achieved, comparable to similar OPDs with evaporated cathode.

Figure 6 shows the Current Density vs. Voltage (JV) characteristics of the photodiode in dark conditions and under AM1.5 illumination. The blue curve is relative to an OPD where the Ag electrode has not been modified throughout the PEI deposition, while the red curve is relative to an OPD with PEI modification, whose structure is depicted in Figure 3. The comparison of the JV characteristics clearly shows a substantial difference in the dark current (more than two orders of magnitude), associated with an overall instability in time, which is reflected in the noisy and irregular shape of the characteristic curve. On the other hand, the characteristic curve of the photodiode with the PEI layer is well aligned to photodetectors obtained with similar material stacks [24,25,26,36]. Finally, Figure 7 shows the EQE under a bias of −4 V of such a device, which results in being essentially flat in the wavelength range between 400 nm and 600 nm, with a maximum value of 70%. The responsivity of this sensor is equivalent to other photodetectors in the literature [26,37].

### 3.3. Tag Functioning

Finally, the whole tag has been exposed to different levels of illumination and the readout of the sensor has been acquired 30 times consecutively. Table 1 represents the obtained ADC counts for each particular conditions as mean value with its standard deviation. In all cases, reference voltages and feedback resistor values of the SFE have been optimized in order to cover the maximum dynamic range of the ADC, 0–1023 counts. Those particular configurations are indicated in Table 1. Two main configurations have been tested to reflect two different real world conditions. First, the exposure to a full sun (AM1.5) has been considered, showing that the photodiode is responsive and its current can be easily detected by the RFID chip. This kind of illumination can be predominant into natural environments; however, it does not cover every typical range of application. In fact, a more realistic and cost-effective approach to detect light permeability of packages could involve the exposure of the parcel to a low-intensity light integrated into the shipping chain. In this second case, a monochromatic light (wavelength 600 nm to match the intensity peak of the EQE of the photodiode) has been shone on the photodetector, with different power densities in the range of some tens of µW (20–60 µW). A proper adjustment of the feedback resistor led to a clear detection of the light and a net discernment between the dark and the illuminated conditions, leading to stable measurements with a low dispersion. Indeed, the tag is capable of not only detecting exposure to light, but also obtaining the absolute luminance level of this exposure.

**Figure 8:**
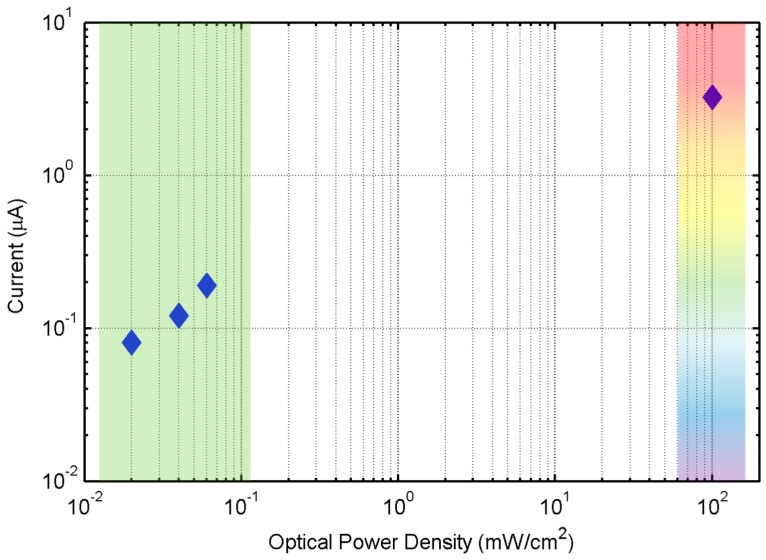
Optical power density vs. photocurrent.

Noticeably, all of these measurements were performed in a solar-cell operation regime for the OPD, namely, without any external bias applied, in order to guarantee the readability of the chip in passive mode and without any need for energy accumulators. Figure 8 represents the obtained photocurrent with respect to the optical power density. The sensing resolution for the OPD is circa 247 µW/cm^2^ in high optical power density mode (*R_fb_* = 185 kΩ) and circa 1.3 µW/cm^2^ in low power density mode (*R_fb_* = 3875 kΩ).

Finally, the tag was introduced in the climatic chamber varying the temperature from 30 °C to 20 °C in 10 min at Relative Humidity (RH) room conditions. The temperature measurements extracted from the tag were in agreement with the chamber sensor within a 0.5 °C difference, which is covered by their respective uncertainties.

## 4. Conclusions

A smart RFID tag fully printed on flexible substrate has been designed, fabricated and tested. The device is comprised of, in an area of 25 cm^2^, a dipole antenna, an SMD inductor, an RFID chip integrating one temperature sensor and a printed photodiode. The RFID tag is able to provide two different environmental parameters: light intensity and temperature. Moreover, sensor information is processed and digitalized on the RFID chip ensuring reliability. Sensor information can be acquired by a commercial EPC Gen 2 RFID reader or be stored on RFID chip EEPROM memory for future access. Therefore, this device is quite interesting for application as supply chain control or environmental light monitoring. The key point of this work is to successfully combine the sprayed photodetector on a flexible substrate, using as the bottom electrode an inkjetted silver line. To succeed in the fabrication process, it was necessary to spray a PEI thin layer on top of the inkjetted silver to obtain a working photodiode.

Regarding sensor performance, the range of detected current can be chosen in the user application. In the present work, we have tested two different ranges of illuminance intensity: 0–100 mW/cm^2^ and 0.02–0.06 mW/cm^2^. In both modes, the results are satisfactory. The device is able to detect currents from hundreds of nA to several µA. In the worst case, the error has been of 10 Least Significant Bits (LSB) that is equivalent to a standard deviation of ±20 nA.

The presented architecture can be applied to other printed sensors and is able to integrate more printed sensors on the same tag. Moreover, single/multiple RFID tags could be attached to different objects or placed in environments where sensor information is necessary. These characteristics, combined with the low production cost of the device and the high possible production throughput, represent a major step towards the realization of low power wireless sensor networks and the growth of the Internet of Things.

## Figures and Tables

**Table 1:** Sensor performance in the RFID tag.

Device/Configuration	Optical Power Density (mW/cm^2^)	ADC Count	Photocurrent (µA)
Sun simulator *R_fb_* = 185 kΩ V_ref1_ = 0 mV V_ref2_ = 410 mV	0	0 ± 0	Below 1.5
	100	810 ± 1	3.23
Monochromator (600 nm) *R_fb_*= 3875 kΩ V_ref1_ = 0 mV V_ref2_ = 460 mV	0.02	0 ± 0	Below 0.8
	0.04	256 ± 8	0.12
	0.08	884 ± 10	0.19
